# Non-specific irreversible ^89^Zr-mAb uptake in tumours: evidence from biopsy-proven target-negative tumours using ^89^Zr-immuno-PET

**DOI:** 10.1186/s13550-024-01079-5

**Published:** 2024-02-15

**Authors:** Jessica E. Wijngaarden, Yvonne W. S. Jauw, Gerben J. C. Zwezerijnen, Berlinda J. de Wit-van der Veen, Daniëlle J. Vugts, Josée M. Zijlstra, Guus A. M. S. van Dongen, Ronald Boellaard, C. Willemien Menke-van der Houven van Oordt, Marc C. Huisman

**Affiliations:** 1 Department of Radiology and Nuclear Medicine, Amsterdam UMC Location Vrije Universiteit Amsterdam, De Boelelaan 1117, Amsterdam, The Netherlands; 2 Imaging and Biomarkers, Cancer Center Amsterdam, Amsterdam, The Netherlands; 3 Department of Haematology, Amsterdam UMC Location Vrije Universiteit Amsterdam, De Boelelaan 1117, Amsterdam, The Netherlands; 4 Department of Nuclear Medicine, Antoni Van Leeuwenhoek Nederlands Kanker Instituut, Plesmanlaan 121, Amsterdam, The Netherlands; 5 Department of Medical Oncology, Amsterdam UMC Location Vrije Universiteit Amsterdam, De Boelelaan 1117, Amsterdam, The Netherlands

**Keywords:** Positron emission tomography, Monoclonal antibodies, Zirconium-89, Molecular imaging

## Abstract

**Background:**

Distribution of mAbs into tumour tissue may occur via different processes contributing differently to the ^89^Zr-mAb uptake on PET. Target-specific binding in tumours is of main interest; however, non-specific irreversible uptake may also be present, which influences quantification. The aim was to investigate the presence of non-specific irreversible uptake in tumour tissue using Patlak linearization on ^89^Zr-immuno-PET data of biopsy-proven target-negative tumours. Data of two studies, including target status obtained from biopsies, were retrospectively analysed, and Patlak linearization provided the net rate of irreversible uptake (*K*_*i*_).

**Results:**

Two tumours were classified as CD20-negative and two as CD20-positive. Four tumours were classified as CEA-negative and nine as CEA-positive. *K*_*i*_ values of CD20-negative (0.43 µL/g/h and 0.92 µL/g/h) and CEA-negative tumours (mdn = 1.97 µL/g/h, interquartile range (IQR) = 1.50–2.39) were higher than zero. Median *K*_*i*_ values of target-negative tumours were lower than CD20-positive (1.87 µL/g/h and 1.90 µL/g/h) and CEA-positive tumours (mdn = 2.77 µL/g/h, IQR = 2.11–3.65).

**Conclusion:**

Biopsy-proven target-negative tumours showed irreversible uptake of ^89^Zr-mAbs measured in vivo using ^89^Zr-immuno-PET data, which suggests the presence of non-specific irreversible uptake in tumours. Consequently, for ^89^Zr-immuno-PET, even if the target is absent, a tumour-to-plasma ratio always increases over time.

**Supplementary Information:**

The online version contains supplementary material available at 10.1186/s13550-024-01079-5.

## Introduction

Positron emission tomography (PET) with zirconium-89-labelled monoclonal antibodies (^89^Zr-mAbs), known as ^89^Zr-immuno-PET, is a powerful tool in precision medicine and drug development. It enables the visualization and quantification of ^89^Zr-mAbs tumour uptake in vivo [1, 2]. These ^89^Zr-immuno-PET studies are generally proof of concept and phase 1 studies, focusing on tracer pharmacokinetics and dose optimization [1]. An important step in the interpretation of PET imaging is understanding the molecular processes underlying the ^89^Zr-mAb uptake on PET.

**Fig. 1:**
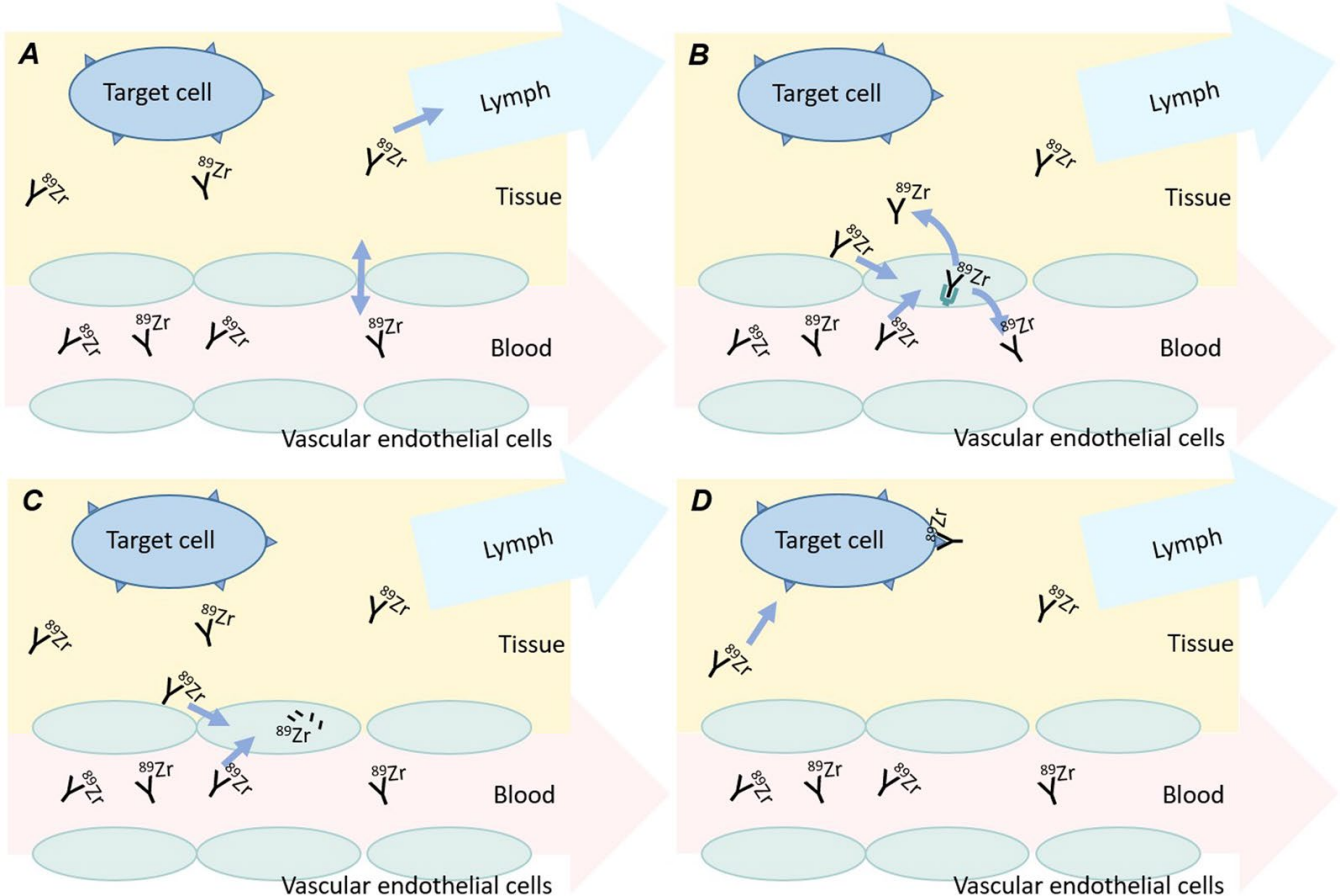
Distribution of ^89^Zr-mAbs in healthy tissue: **A** convection of mAbs across the vascular endothelium into the interstitial space and return to circulation via lymphatic system; **B** mAb uptake into endothelial cells via pinocytosis and FcRn-mediated return to vascular or interstitial space; **C** catabolism of FcRn-unbound mAbs; **D** binding to target receptors. Based on Lobo et al. [4]

Distribution of mAbs into tissue depends on the structure of the capillary endothelium [3] and can occur via different processes. Firstly, mAbs distribute across the vascular endothelium into the interstitial space, and leave the interstitial space with lymph fluid, both via convective transport [3, 4] (see Fig. 1). Secondly, transport may take place via diffusion. Diffusion is limited in healthy tissue due to the relatively large size of mAbs [4], but it may play a more significant role in tumour tissue. Due to rapid cell division, the endothelial cells in tumour vasculature are often disorganized leading to leakiness [3]. The increased permeability allows diffusion of mAbs across the tumour endothelium and, together with the poor lymph drainage in tumour tissue, leads to accumulation inside the interstitial space (i.e. the enhanced permeability and retention (EPR) effect) [5]. Thirdly, mAbs may enter endothelial cells via receptor-mediated endocytosis where they can bind to the neonatal Fc-receptor (FcRn) [4] (see Fig. 1). Subsequently, FcRn-bound mAbs are brought back into the blood circulation or interstitial space, while FcRn-unbound mAbs are catabolized by lysosomes [4]. This catabolism is the main elimination pathway for mAbs [4, 6]. Literature is unequivocal about the role of FcRn in tumour tissue. FcRn has been shown to be present at very low levels in most human tumour cell lines [7] and was described to be downregulated and dysregulated in various cancer types [8, 9]. Both dysregulation and upregulation of FcRn were described to be associated with tumour growth [8, 10]. Moreover, mAbs may bind to Fcγ receptors on immune cells [11]. Lastly, mAbs bind to the specific target receptor present within the tissue (see Fig. 1).

The different distribution processes may contribute to the ^89^Zr-mAb uptake measured on PET [12]. Firstly, the distribution of mAbs into the interstitial space (via convection, diffusion or FcRn binding) and subsequent re-circulation via the lymphatic system results in non-specific reversible ^89^Zr-mAb uptake. Secondly, FcRn-unbound mAbs are catabolized within endothelial cells leading to residualization and off-target accumulation of ^89^Zr [13], referred to as non-specific irreversible uptake [12]. Binding of mAbs to Fcγ receptors may also contribute to non-specific irreversible uptake, as studied previously [11]. Thirdly, actual target-specific binding takes place, which leads to target-specific irreversible ^89^Zr-mab uptake for irreversibly binding or internalizing tracers. As the non-specific irreversible ^89^Zr-mAb uptake is inversely related to FcRn binding, the presence of this uptake mechanism may provide insight into the role of FcRn in tumour tissue.

The reason for our interest in non-specific irreversible uptake in tumours is twofold. Firstly, uptake on PET is commonly quantified using the standardized uptake value (SUV) [14]. For ^89^Zr-immuno-PET, there is an increased interest in reporting the tumour-to-plasma ratio (TPR), which provides more valid results in quantifying ^89^Zr-mAb uptake when the mass dose is varied as compared to SUV [15]. Target-specific uptake is of main interest, but both measures quantify the total uptake, in which non-specific irreversible and reversible uptake may be present as well. Secondly, a more comprehensive approach to describe the distribution of mAbs is with the use of physiologically based pharmacokinetic (PBPK) models. Some models did incorporate FcRn binding within the tumour compartment [16–18], while in other models, the distribution of mAbs into the tumour interstitial space is determined by the EPR effect [19–23]. PBPK models potentially improve our understanding of ^89^Zr-immuno-PET studies; however, an in-depth understanding the role of FcRn in tumour tissue is lacking but desired [8, 10].

The different contributions of ^89^Zr-mAb uptake to the PET signal have been separated previously using Patlak linearization [12, 24–32]. This method enables separation of PET signal resulting from reversible and irreversible uptake processes [33]. It is assumed that uptake of ^89^Zr-mAbs can be separated in these two components, where irreversible uptake includes both target-specific and non-specific uptake. Patlak linearization was applied to ^89^Zr-immuno-PET data of healthy non-target-expressing organ tissue to establish baseline uptake values representing the non-specific irreversible uptake. As shown by Jauw et al., comparison of uptake in target-expressing organs with these baseline values allows quantification of actual target-specific uptake [12]. Additionally, when applying the co-infusion of additional doses of unlabelled mAbs, the competition in binding between ^89^Zr-labelled and unlabelled mAbs has shown to lead to a decreased uptake of ^89^Zr-labelled mAb. This enables assessment of target engagement in tumour tissue [24].

A similar approach of measuring uptake of ^89^Zr-mAbs in non-target-expressing tissue was applied here to tumour tissue to study the role of non-specific irreversible uptake. The current study aimed to investigate the presence of non-specific irreversible uptake in tumour tissue using Patlak linearization on ^89^Zr-immuno-PET data of biopsy-proven target-negative tumours. Such a dataset is quite unique and is based on incidental findings, because applying unspecific antibody in a clinical setting is generally not ethically justified. An estimation of the contribution of non-specific irreversible uptake in tumour tissue will enable the quantification of actual target-specific ^89^Zr-mAb uptake.

## Methods

### Data overview

Data of two studies, in which biopsied tumour data concerning the target status was available, were retrospectively analysed (NTR3392, NCT02004106). Seven patients with diffuse large B-cell lymphoma received a therapeutic dose of anti-CD20 mAb (range 700–1000 mg) followed by 10 mg 74 MBq ^89^Zr-anti-CD20 (for additional information see [34]). Three PET/CT scans were obtained 1–2 h, 3 days (72–78 h) and 6/7 days (140–166 h) p.i., and blood samples were drawn up to four times within 3 h after tracer administration and with every PET scan. The following data are needed for the purpose of the current study: PET scans with accompanying blood samples at two or more time points after ~ 24 h p.i., and at least one blood sample in the first hours after injection for calculation of the area under the plasma curve. For Patlak linearization, an equilibrium between plasma tracer concentration and unbound tracer in tissue is required, which is assumed to be reached ~ 24 h p.i. for mAbs [6, 12, 35, 36]. Therefore, two patients were excluded because of missing blood samples and one patient was excluded because of a missing PET scan. This resulted in evaluable data of four patients.

Twenty-four patients with metastatic solid malignancies (i.e. colorectal cancer, non-small cell lung cancer, salivary gland cancer, gastric cancer) received 6, 20 or 30 mg anti-CEA-IL2v of which 2 mg containing 50 MBq ^89^Zr-CEA-IL2v [37]. Anti-CEA-IL2v is a bispecific mAb targeting carcinoembryonic antigen (CEA), which also contains an IL2v moiety that binds to IL-2 receptor β and γ, but with abolished IL-2 receptor α binding [38]. Though the study design consisted of two tracer administrations, only the ^89^Zr-immuno-PET scans prior to the first treatment cycle were selected for the current study. PET/CT scans were scheduled at 2 h, 1 day (20–27 h), 4 days (92–100 h) and 8 days (189–193 h) p.i., and blood samples were drawn three times within 4 h p.i. and with every PET scan. Of the 24 patients, data of one patient were excluded, since there was no biopsy available because the tumour was [^18^F]-FDG-PET-negative [37], seven patients were excluded because the exact location of the biopsy was uncertain, one patient was excluded because of a missing blood sample, one patient was excluded because of unreliable blood sampling data, which resulted in 14 evaluable patients. An overview of the patient inclusion for both studies is shown in Additional file 1: Figure S1.

### Immunohistochemistry

As part of the original study designs, biopsies were taken from one tumour lesion for each patient. Biopsied tumour lesions were identified on [^18^F]-FDG PET. Following routine clinical procedure, CD20 or CEA expression was assessed using immunohistochemistry (IHC). For this study, tumours were classified as either present (positive) or completely absent (negative). CD20 expression was completely absent in both CD20-negative tumour lesions (IHC results of one of them are presented in [34]). From one of the patients, a second biopsy was taken at a different location within the same tumour lesion, which confirmed the absence of CD20 expression. Also in the CEA-negative tumour lesions, expression was completely absent, and CEA-negative was defined as 0% staining [37]. It is important to note that there was no IL-2Rβγ expression status available, regardless of the CEA expression status.

### Tumour delineation

The biopsied tumours (with volumes > 1 mL) were identified on the PET scans by a nuclear medicine physician. Tumours were delineated using the ACCURATE tool [39]*.* Visually positive tumours (i.e. lesion uptake higher than background) on ^89^Zr-immuno-PET were manually delineated, and peak activity concentration values [Bq/mL] were obtained. Visually negative tumours on ^89^Zr-immuno-PET were manually delineated based on the low dose-CT (ldCT). Mean activity concentration values were obtained, because peak activity concentration would be more prone to error in case of low tumour uptake. One tumour was not visible on PET and ldCT; therefore, the location was determined based on surrounding tissue and a 3-dimensional spherical region of interest (of 2.1 mL) was placed.

### Patlak linearization

Patlak linearization enables separation of reversible and irreversible uptake [33]. For this purpose, activity concentration in tumour and plasma on two or more time points is required. In addition, an equilibrium between plasma concentration and unbound tracer in tumour tissue is required, which is assumed to be reached ~ 24 h p.i. for mAbs [6, 12, 35, 36]. The obtained activity concentration data can be presented in a Patlak plot according to the Patlak equation. Graphical analysis of the Patlak plot, assuming a linear relationship, provides the slope (*K*_*i*_), representing the net influx rate of irreversible uptake [µL/g/h], and the offset (*V*_*T*_), approaching the total distribution volume, as a measure for the reversible part [33]. Patlak linearization was applied to determine the *K*_*i*_ value for each tumour. Data from one tumour of the ^89^Zr-CEA-IL2v study were excluded because of variation in the Patlak linearization (*R* < 0.9), resulting in inclusion of data of seventeen tumours. Here we assumed, as previously [12], that data for ^89^Zr-anti-CD20 were consistent with the assumptions of Patlak linearization, because irreversible uptake of tracer is expected. Therefore, Patlak linearization was applied even though there were only two data points available for the ^89^Zr-anti-CD20 study.

## Results

For five patients who received ^89^Zr-anti-CEA-IL2v, one PET scan was missing, and for three patients, one blood sample was missing. Therefore, for obtaining the Patlak *K*_*i*_ values, three imaging time points were included in five cases, and two time points in twelve cases.

Of the patients who received ^89^Zr-anti-CD20, two tumours were classified as CD20-negative and two were CD20-positive according to IHC. Of the patients who received ^89^Zr-anti-CEA-IL2v, four tumours were classified as CEA-negative and nine tumours were CEA-positive.

**Fig. 2:**
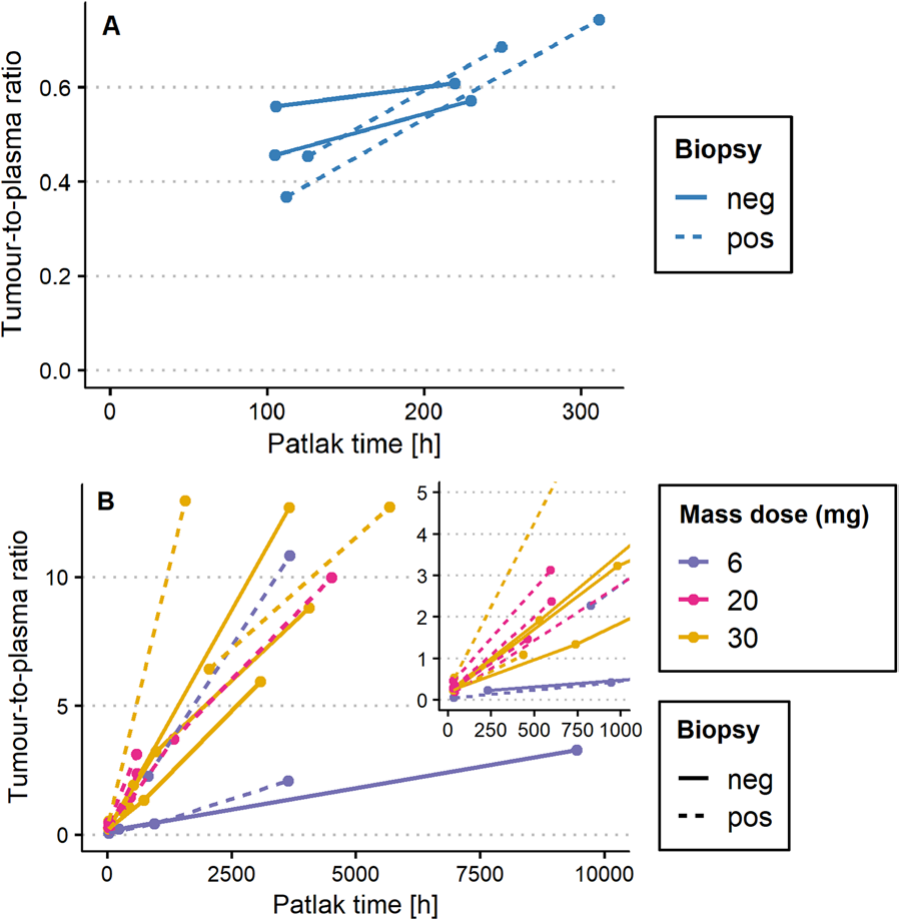
Individual Patlak linearization plots from the ^89^Zr-anti-CD20 study (**A**) and the ^89^Zr-CEA-IL2v study (**B**). neg = target-negative, pos = target-positive

**Fig. 3:**
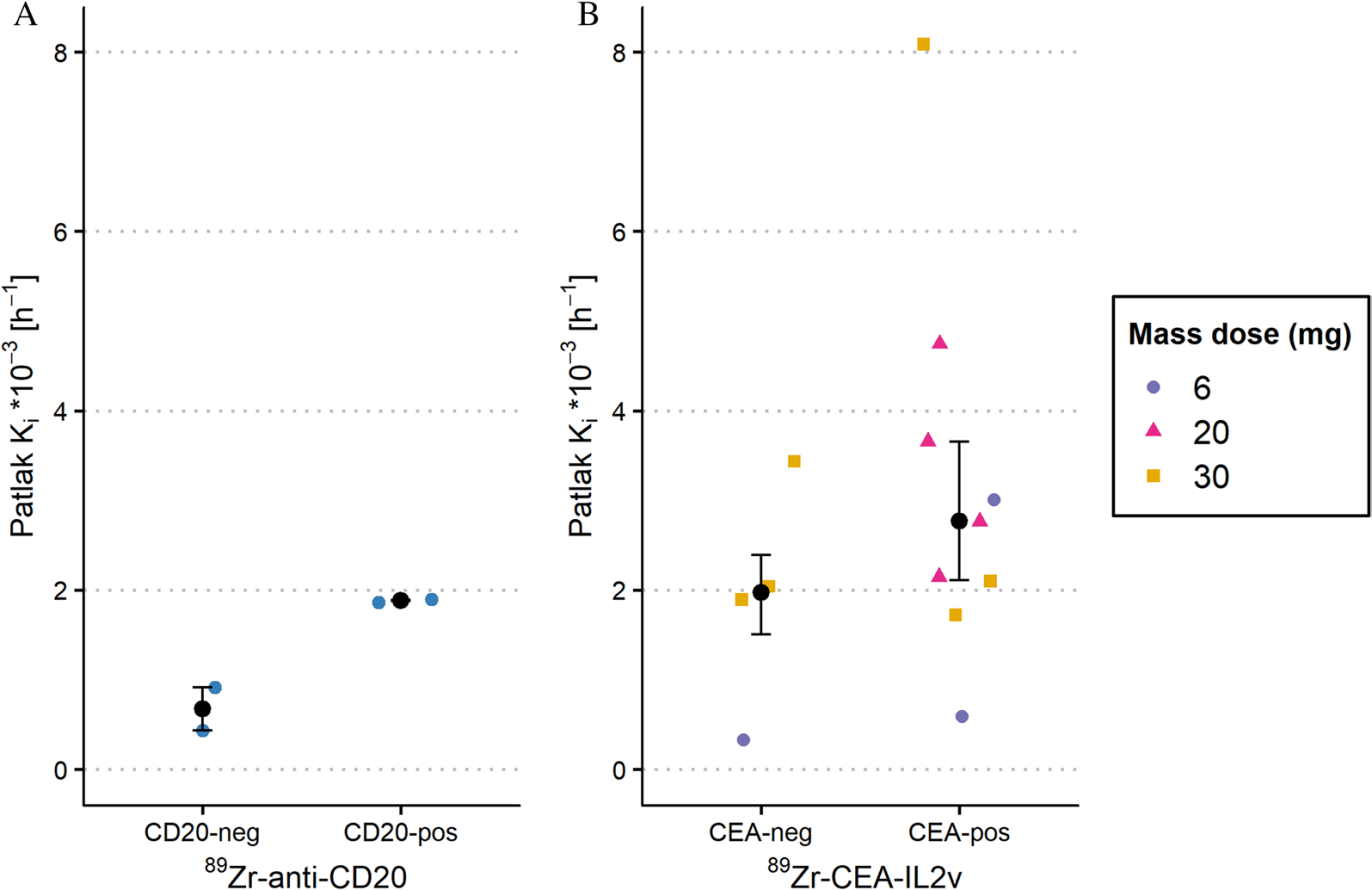
Patlak *K*_*i*_ values, representing irreversible uptake, of biopsy-proven target-negative and target-positive tumours from the ^89^Zr-anti-CD20 study (**A**) and the ^89^Zr-CEA-IL2v study (**B**). Median and interquartile range values are presented in black

Individual Patlak plots are presented in Fig. 2. From these Patlak plots, the *K*_*i*_ values (i.e. the slopes) were obtained. *K*_*i*_ values of both CD20-negative (0.43 µL/g/h and 0.92 µL/g/h) and CEA-negative tumours (mdn = 1.97 µL/g/h, interquartile range (IQR) = 1.50–2.39) were higher than zero (see Fig. 3). The median *K*_*i*_ values of CD20-positive tumours (1.87 µL/g/h and 1.90 µL/g/h) and CEA-positive tumours (mdn = 2.77 µL/g/h, IQR = 2.11–3.65) were higher than those of target-negative tumours (see Fig. 3). A complete overview of the data is shown in Additional file 1: Tables S1 and S2.

## Discussion

The presence of non-specific irreversible uptake in tumour tissue was studied by applying Patlak linearization on ^89^Zr-immuno-PET data of biopsy-proven target-negative tumours. Biopsy-proven target-negative tumours showed positive *K*_*i*_ values suggesting the presence of non-specific uptake of ^89^Zr-mAbs measured in vivo using ^89^Zr-immuno-PET. Additionally, as expected, the irreversible uptake in target-positive tumours was higher than in target-negative tumours. Moreover, irreversible uptake of ^89^Zr-CEA-IL2v was higher than for ^89^Zr-anti-CD20.

Despite the absence of target expression in the target-negative tumours, there was still irreversible uptake found, i.e. a *K*_*i*_ value above zero. This non-specific irreversible uptake in tumours is most likely due to the degradation of ^89^Zr-mAbs that are not protected by FcRn. Also, binding of mAbs to Fcγ receptors on immune cells may play a role in irreversible ^89^Zr-mAb uptake [11]. Previous literature, however, describes that extravasation of mAbs into the tumour interstitial space occurs primarily via diffusion [20]. The increased permeability and poor lymphatic drainage in tumour tissue lead to the EPR effect, resulting in accumulation of mAbs within the interstitial space [5, 40]. Nonetheless, this diffusion process would result in reversible uptake, as it does not include degradation of mAbs. The current study suggests the presence of non-specific irreversible uptake, which contradicts the conception that extravasation occurs solely via diffusion. Both protection by FcRn and the EPR effect are thought to contribute to ^89^Zr-mAbs extravasation into tumour tissue; however, the ratio between them may differ considering the high intra-tumour variability in characteristics such as vascularization, morphology, level of necrosis, size, acidity and immunogenic potential [41–43].

PET imaging has been used previously to provide a measurement for non-specific ^89^Zr-mAb uptake in healthy tissue. Jauw et al. analysed ^89^Zr-immuno-PET data of four different ^89^Zr-mAbs in organs (i.e. lungs, liver, kidney and spleen) in which target expression was absent. *K*_*i*_ values ranged from 0.2 µL/g/h for lung to 1.1 µL/g/h for liver, representing the rate of catabolism of ^89^Zr-mAbs not protected by FcRn [12]. These values are comparable to the values for non-specific irreversible uptake in CD20-negative tumours found in this study. A *K*_*i*_ value of zero would be expected in tissue where neither target-specific nor non-specific uptake is present. For example in brain tissue, where the blood–brain barrier hampers ^89^Zr-mAb distribution, a Patlak *K*_*i*_ value of 0.0 to 0.1 µL/g/h was recently found [28], which suggests that the values found in the current study are not merely variability in data.

Both the target-positive and target-negative tumours of the ^89^Zr-CEA-IL2v data showed higher irreversible ^89^Zr-mAb uptake than the ^89^Zr-anti-CD20 data. Additionally, the *K*_*i*_ value in CEA-negative tumours was also slightly higher than previously found in healthy organs without target expression [12]. This is likely the result of binding of ^89^Zr-CEA-IL2v to IL-2Rβγ receptors on immune cells [38]. CEA-IL2v has shown to bind to CD8 + T and natural killer cells and leads to the expansion of these cells in the blood, lymphoid tissue and tumours [38]. Binding to these tumour-infiltrating immune cells would result in increased *K*_*i*_ values when these ^89^Zr-CEA-IL2v-bound immune cells remain inside the tumour tissue during the course of the study. On the other hand, in a previous study, the concentrations of CEA-IL2v in blood and the uptake in tumour were predicted using a mathematical model [44]. In this model, only the unbound CEA-IL2v was used as input for tumour uptake, and not the complex of CEA-IL2v with IL-2Rβγ expressed on immune cells [44]. Nonetheless, the binding of ^89^Zr-CEA-IL2v to immune cells would explain the difference in irreversible binding with the ^89^Zr-anti-CD20 study.

In conventional PET pharmacokinetic modelling, dynamic PET imaging data are obtained and fitted to different one- and two-tissue models [45]. The best model fit indicates the pharmacokinetic characteristics of the tracer. For a tracer that is best described by a two-tissue irreversible model, Patlak linearization is a simplified approach to obtain the pharmacokinetic constant for irreversible uptake, *K*_*i*_. In ^89^Zr-immuno-PET, static PET images are acquired at multiple days p.i. because of the long half-life of mAbs. This leads to a limited amount of data for which conventional modelling is not possible. Instead, Patlak linearization is applied to ^89^Zr-immuno-PET studies, assuming a two-tissue irreversible model. PBPK modelling is a different type of modelling that considers multiple tissues represented by different compartment. Contrary to conventional pharmacokinetic modelling, PBPK modelling is not data-driven, but allows a priori predictions of tracer concentrations based on physiological information [16]. This allows evaluation of the separate processes that are involved in mAb pharmacokinetics. All quantification approaches assume that the processes between compartments occur at a constant rate during the course of the study. However, migration of immune cells might potentially influence quantification of PET imaging, which should be kept in mind for the ^89^Zr-CEA-IL2v study, as discussed previously.

There are some limitations to the study. Ideally, Patlak linearization would be applied to three imaging time points with one time point at 24 h p.i, as this improves the accuracy and precision of the method [35]. It is important to note that in this study, there were only two imaging time points available in twelve out of seventeen cases. Additionally, the ^89^Zr-anti-CD20 data did not include an imaging time point around 24 h p.i. From simulation studies, it can be seen that the *K*_*i*_ value is always slightly underestimated, even more so when there is no 24 h p.i. time point included. Therefore, an underestimation of the *K*_*i*_ value of about 8% may be expected [35]. Additionally, the current study evaluates ^89^Zr-mAb uptake in target-negative tumours, which is a unique dataset, but consequently also a limited amount of data. In future studies, a *K*_*i*_ value for non-specific irreversible uptake in tumours may be determined more reliably, and its translation to other ^89^Zr-mAbs may be evaluated.

^89^Zr-mAb tumour uptake on PET was interpreted in the context of the target presence measured with IHC. There are aspects to consider when relating PET uptake to IHC. IHC is currently the standard procedure in the clinic for measuring target expression. However, the biopsy obtained for IHC assessment is a small part of the tumour and sampling errors may occur, while tumour uptake on PET is obtained from the whole tumour. Heterogeneity in target expression within the whole tumour will therefore not be detected with IHC. However, CEA-negative tumours are thought to be highly unlikely to have expressed any CEA [37, 46]. Additionally, one of the CD20-negative tumours was biopsied twice, both showing no target expression.

The current study was performed using data of patients with two different types of cancer. DLBCL develops from the B cells in the lymphatic system and is characterized by CD20 expression, which is a transmembrane protein [34]. The anti-CD20 mAbs target the malignant B cells and trigger cell death. For the majority of solid malignancies, amongst which also colorectal, non-small cell lung and gastric cancer, CEA is overexpressed [37, 38]. The CEA-IL2v bispecific mAbs induce the local immune response by binding to the CEA in tumour tissue while also binding to immune cells. The difference between these two mAbs is in their variable region; however, their constant region is similar. Since the constant region binds to FcRn [4], no differences in uptake of target-negative tumours may be expected.

During the two clinical studies, different mass doses were applied which is known to influence target-specific ^89^Zr-mAb uptake. Administration of additional unlabelled mAbs results in competition between ^89^Zr-labelled and unlabelled mAbs for target binding [24]. Because of this competition and the limited number of receptors, there is less target-specific uptake resulting in lower *K*_*i*_ values. Three different mass doses of ^89^Zr-CEA-IL2v were administered to the patients: 6, 20 or 30 mg. The mass dose of 30 mg indeed resulted in lower *K*_*i*_ values in target-positive tumours as compared to the 20 mg mass dose. However, the mass dose of 6 mg resulted in lower *K*_*i*_ values compared to the higher mass doses. The reason for this is unknown, a possible explanation might be the spleen acting as a IL-2Rβγ sink, as has been previously hypothesized by [37]. This results in low plasma activity concentrations and less supply for the tumour. Nonetheless, non-specific uptake is not influenced by differences in mass dose, because it results from degradation of mAbs not protected by FcRn, a process that is not saturable with these relatively low mass doses [4]. Since the irreversible uptake in target-negative tumours only results from this non-specific mAb degradation, the *K*_*i*_ values in target-negative tumours are not influenced by differences in mass dose.

The current study shows that non-specific irreversible uptake is present in the absence of specific targets. This contribution may interfere with the quantification of actual target-specific irreversible uptake, which is of interest. If a baseline value for non-specific irreversible tumour uptake could be established in future, it may be used as a correction when quantifying ^89^Zr-mAb uptake in tumours, similar to what was previously proposed by Jauw et al. to use baseline *K*_*i*_ values in healthy tissue to measure target engagement [12]. It is, however, more common to report SUV or TPR. Since the *K*_*i*_ value represents the rate of irreversible uptake, a positive *K*_*i*_ value means that a TPR always increases over time. The Patlak linearization described in this manuscript can be used to estimate the contribution of non-specific irreversible uptake to a SUV or TPR (see Additional file 1: Figure S2). For ^89^Zr-anti-CD20 uptake at the latest imaging time point, the total uptake would consist of 71% irreversible uptake, of which the larger part is CD20-specific uptake (46%) and the smaller part is non-specific uptake (25%). For the ^89^CEA-IL2v study, the total uptake at the latest imaging time point would be dominated by irreversible uptake (94%). This is separated into CEA-specific uptake (27% of total uptake) and non-specific uptake (67% of total uptake), which most likely includes irreversible binding to IL-2βγ receptors [38]. Consequently, both specific and non-specific irreversible uptakes are highest at the latest imaging time point. PET scans should be scheduled as late as possible, while still acquiring reliable PET scans considering the decreasing number of counts over time due to radioactive decay. Because of this trade-off, PET scans are typically acquired up to seven days p.i. [2].

## Conclusion

The aim of this study was to investigate the presence of non-specific irreversible ^89^Zr-mAbs uptake in tumours, as it may interfere with quantification of target-specific uptake. Patlak linearization was retrospectively applied to ^89^Zr-immuno-PET data of biopsy-proven target-negative tumours to measure the irreversible uptake (expressed in a *K*_*i*_ value). Biopsy-proven target-negative tumours showed Patlak *K*_*i*_ values above zero, which suggests the presence of non-specific irreversible uptake. The Patlak *K*_*i*_ values in the current study are consistent with non-specific irreversible uptake in healthy organs. Consequently, a tumour-to-plasma ratio always increases over time for ^89^Zr-mAbs uptake in tumours, also in absence of the target.

### Supplementary Information

**Additional file 1: **Additional figures and analyses.

## Data Availability

The datasets analysed during the current study are available from the corresponding author on reasonable request.
