# Supplementary material for: Non-specific irreversible 89Zr-mAb uptake in tumours: evidence from biopsy-proven target-negative tumours using 89Zr-immuno-PET
Source: EJNMMI Res. 2024 Feb 15;14:18. doi: 10.1186/s13550-024-01079-5 (PMC10869322; doi:10.1186/s13550-024-01079-5)
Supplement: Supplementary file 1 — Additional file 1: Additional figures and analyses. [file 13550_2024_1079_MOESM1_ESM.docx]

**Supplemental material**


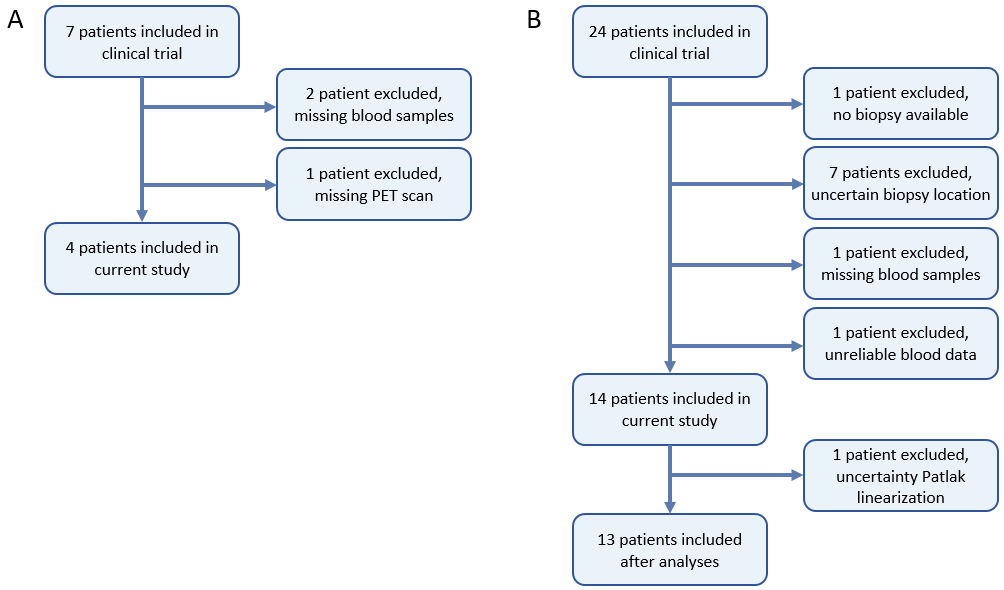


**Figure S1**: Flow chart of included patients from the ^89^Zr-anti-CD20 study *(1)* (**A**) and the ^89^Zr-CEA-IL2v study *(2)* (**B**).

**Table S1**: Data overview of the ^89^Zr-anti-CD20 study *(1)*.

| **Patient** | **Mass dose (mg)** | **Tumour location** | **CD20 expression** | **^89^Zr-mAb PET** | **Ki ·10^-3^ (h^-1^)** |
| --- | --- | --- | --- | --- | --- |
| 1 | 860 | Liver | Negative | Negative | 0.92 |
| 3 | 1010 | Mesenterial | Negative* | Positive | 0.43 |
| 5 | 710 | Retro-peritoneal | Positive | Positive | 1.87 |
| 6 | 1010 | Nasopharynx | Positive | Positive | 1.90 |

* a second biopsy confirmed absence of CD20 expression

**Table S2**: Data overview of the ^89^Zr-CEA-IL2v study *(2).*

| **Patient** | **Mass dose (mg)** | **Tumour location** | **CEA expression** | **^89^Zr-mAb PET** | **Ki ·10^-3^ (h^-1^)** |
| --- | --- | --- | --- | --- | --- |
| 6001 | 6 | Supraclavicular LN | Positive | Positive | 3.00 |
| 6002 | 6 | Omentum | Negative | Negative | 0.33 |
| 6003 | 6 | Subcutaneous (abdomen) | Positive | Negative | 0.58 |
| 6011 | 20 | Lung | Positive | Negative | 2.15 |
| 6031 | 20 | Lung | Positive | Positive | 3.65 |
| 6032 | 20 | Parotid | Positive | Positive | 4.76 |
| 6033 | 20 | Caecum | Positive | Positive | 2.77 |
| 6006 | 30 | Para umbilical LN | Negative | Positive | 3.42 |
| 6007 | 30 | Supraclavicular LN | Positive | Positive | 8.09 |
| 6008 | 30 | Mediastinal LN | Positive | Positive | 1.73 |
| 6009 | 30 | Subcutaneous (shoulder) | Negative | Negative | 1.89 |
| 6012 | 30 | Iliac LN | Negative | Positive | 2.05 |
| 6028 | 30 | Lung | Positive | Positive | 2.11 |

**Supplemental analysis**: Estimation of different contributions to ^89^Zr-mAb uptake

Methods:

The data allow to estimate the different contributions for these specific datasets. We simulated the tumor activity concentrations (*AC­_T_*) from the median *K_i_* and *V_T_* values together with the median activity concentration in plasma (*AC_P_*) that were obtained from these datasets. From the Patlak equation: *AC_T_ = AUC_P_*K_i_ + AC_P_ · V­_T_*, we can use *AUC_P_*K_i_* to calculate the irreversible part (*AUC_P_* = area under plasma curve) and *AC_P_*V_T_* to calculate the reversible part. For the reversible part, the *V_T_* values for target-positive and target-negative tumors were averaged. The non-specific irreversible uptake was simulated using the *K_i_* value obtained from target-negative tumors. The total irreversible uptake was simulated using the *K_i_* value obtained from target-positive tumors. The specific irreversible uptake was calculated by subtracting the non-specific from total irreversible uptake. Median values for injected activity and body weight were used to calculate simplified uptake values (SUV_BW_).

Results:


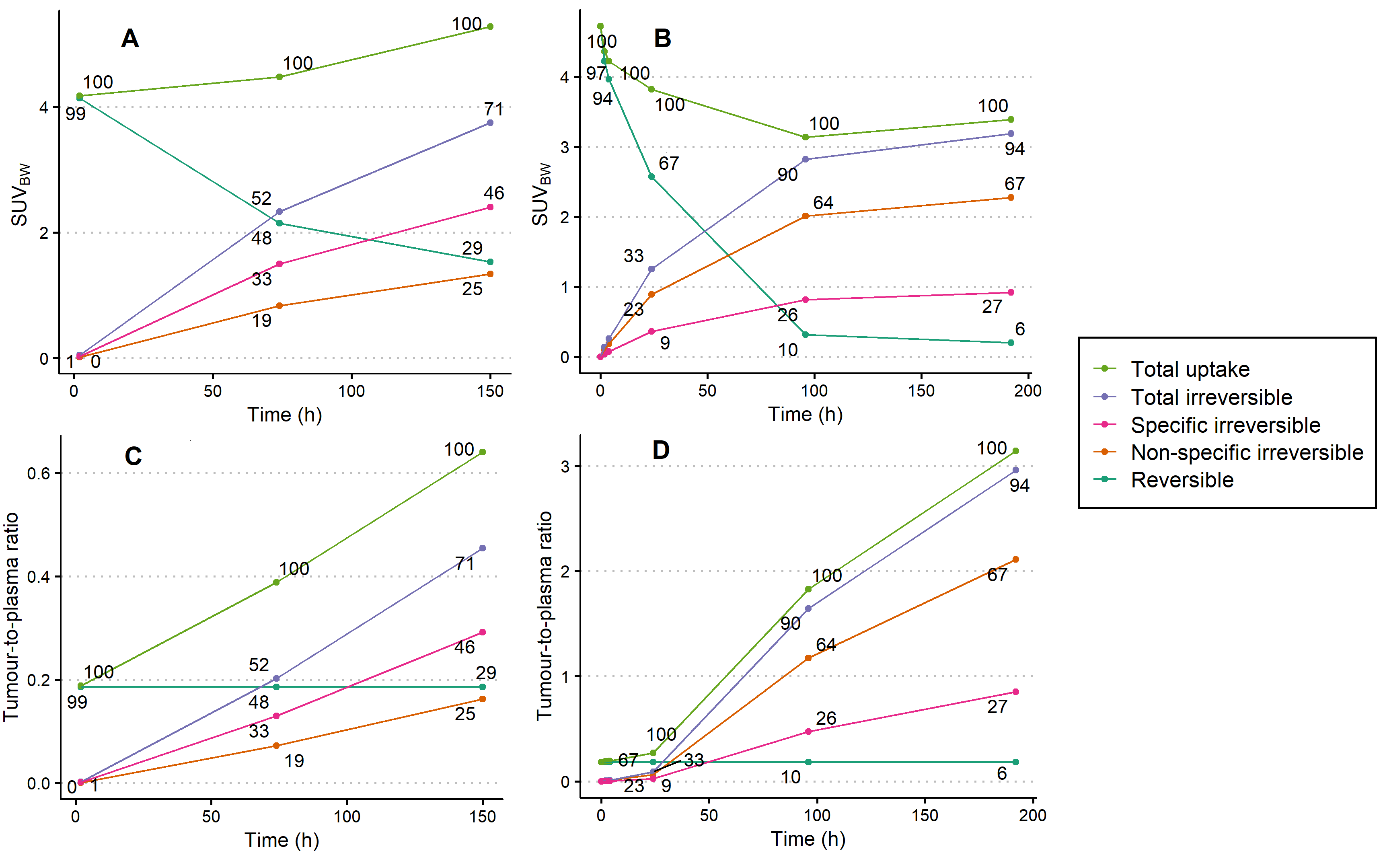


**Figure S2**: Estimation of the different contributions of uptake to a SUV_BW_ (**A, B**) and TPR (**C, D**) and for the ^89^Zr-anti-CD20 study (**A, C**) and the ^89^Zr-CEA-IL2v study (**B, D**), calculated using Patlak linearisation.

At each time point, the contribution of each component (reversible, non-specific irreversible, total irreversible and specific irreversible) was also calculated as percentage of the total uptake and presented in text in the figures. For ^89^Zr-anti-CD20, there is a quite large contribution of reversible uptake (29% at latest time point). This is most likely due to the larger therapeutic mass dose that was applied within this study, which leads to lower PET uptake due to the competition between labelled and unlabelled mAb *(3)*. Of the 71% of irreversible uptake, the larger part is specific uptake (46%) and the smaller part is non-specific uptake (25%). For ^89^Zr-CEA-IL2v, the total uptake at the latest time point is dominated by irreversible uptake (94%). This is separated into CEA-specific uptake (27% of total uptake), and non-specific uptake (67% of total uptake) which possibly includes irreversible binding to IL-2βγ receptors. Consequently, both specific and non-specific irreversible uptake are highest at the latest imaging time point. PET scans should be scheduled as late as possible, while still acquiring reliable PET scans considering the decreasing number of counts over time due to radioactive decay. Because of this trade-off, PET scans are typically acquired up to seven days p.i. *(4)*.

**References**

1. Jauw YW, Zijlstra JM, de Jong D, Vugts DJ, Zweegman S, Hoekstra OS, et al. Performance of 89Zr-Labeled-Rituximab-PET as an Imaging Biomarker to Assess CD20 Targeting: A Pilot Study in Patients with Relapsed/Refractory Diffuse Large B Cell Lymphoma. PLoS One. 2017;12(1):e0169828.

2. van Brummelen EM, Huisman MC, Nayak TK, Stokkel MP, Mulder ER, Hoekstra OS, et al. 89Zr-labeled CEA-targeted IL-2 variant immunocytokine in patients with solid tumors: CEA-mediated tumor accumulation and role of IL-2 receptor-binding. Oncotarget. 2018;9(37):24737.

3. Menke-van der Houven van Oordt CW, McGeoch A, Bergstrom M, McSherry I, Smith DA, Cleveland M, et al. Immuno-PET Imaging to Assess Target Engagement: Experience from (89)Zr-Anti-HER3 mAb (GSK2849330) in Patients with Solid Tumors. J Nucl Med. 2019;60(7):902-9.

4. Jauw YW, Menke-van der Houven van Oordt CW, Hoekstra OS, Hendrikse NH, Vugts DJ, Zijlstra JM, et al. Immuno-Positron Emission Tomography with Zirconium-89-Labeled Monoclonal Antibodies in Oncology: What Can We Learn from Initial Clinical Trials? Front Pharmacol. 2016;7:131.
